# Learned-SBL-GAMP based hybrid precoders/combiners in millimeter wave massive MIMO systems

**DOI:** 10.1371/journal.pone.0289868

**Published:** 2023-09-08

**Authors:** Shoukath Ali K., Arfat Ahmad Khan, Perarasi T, Ateeq Ur Rehman, Khmaies Ouahada

**Affiliations:** 1 Department of Electronics and Communication Engineering, Presidency University, Itgalpura, Rajanukunte, Yelahanka, Bengaluru, Karnataka, India; 2 Department of Computer Science, College of Computing, Khon Kaen University, Khon Kaen, Thailand; 3 Department of Electronics and Communication Engineering, Bannari Amman Institute of Technology, Anna University, Erode, Tamil Nadu, India; 4 Department of Electrical Engineering, Government College University, Lahore, Pakistan; 5 Department of Electrical and Electronic Engineering Science, University of Johannesburg, Johannesburg, South Africa; Mae Fah Luang University, THAILAND

## Abstract

In Millimeter-Wave (mm-Wave) massive Multiple-Input Multiple-Output (MIMO) systems, hybrid precoders/combiners must be designed to improve antenna gain and reduce hardware complexity. Sparse Bayesian learning via Expectation Maximization (SBL-EM) algorithm is not practically feasible for high signal dimensions because estimating sparse signals and designing optimal hybrid precoders/combiners using SBL-EM still provide high computational complexity for higher signal dimensions. To overcome the issues of high computational complexity along with making it suitable for larger data sets, in this paper, we propose Learned-Sparse Bayesian Learning with Generalized Approximate Message Passing algorithm (L-SBL-GAMP) algorithm for designing optimal hybrid precoders/combiners suitable for mmWave Massive MIMO systems. The L-SBL-GAMP algorithm is an extension of the SBL-GAMP algorithm that incorporates a Deep Neural Network (DNN) to improve the system performance. Based on the nature of the training data, the L-SBL-GAMP can design the optimal Hybrid precoders/combiners, which enhances the spectral efficiency of mmWave massive MIMO systems. The proposed L-SBL-GAMP algorithm reduces the iterations, training overhead, and computational complexity compared to the SBL-EM algorithm. The simulation results unveil that the proposed L-SBL-GAMP provides higher achievable rates, better accuracy, and low computational complexity compared to the existing algorithm, such as Orthogonal Matching Pursuit (OMP), Simultaneous Orthogonal Matching Pursuit (SOMP), SBL-EM and SBL-GAMP for mmWave massive MIMO architectures.

## Introduction

During the last few decades, many end users are connected to the internet due to the technological enhancements in communication devices [1, 2]. As a result, lower frequency bands have been congested. To tackle this issue, scientists have explored new ways, such as equipping base stations with massive antennas [3, 4], spectrum coding, and network densification algorithms [5]. These ways have solved the issues; however, they are not enough to meet the ever-increasing demand of users. This situation has shifted the focus of research toward millimeter-wave frequencies (mmWave) [6].

The spectrum of mmWave ranges from 30GHz-300GHz, and it is deemed a perfect candidate to meet the requirements of 5G networks, as they require higher spectral and energy efficiencies [7]. However, the main issues associated with mmWave frequencies are related to high path losses and severe blockages [8]. It has been seen that the large antenna array at the base station helps to significantly reduce high path losses [9]. Meanwhile, a large antenna array can easily be installed at the base station owing to the small wavelength of mmWave frequencies. Therefore, the incorporation of mmWave with large antenna arrays at the base station is one of the most suitable options to fulfill the requirements of a current and future generation of communication networks. A large antenna array at the base station is another promising wireless technology to fulfill the requirements of spectral and energy efficiencies for the current and future wireless communication systems [10].

Contrary to the conventional MIMO systems [11] where each transceiver chain is equipped with the individual Digital to Analogue Convertor (DAC), Analogue to Digital Convertor (ADC), frequency matches, and amplifiers, researchers opt for hybrid architectures in mmWave communication systems to reduce the cost. In hybrid architecture, the precoders and combiners are designed by a combination of analog and digital precoders and combiners for processing the transmitting and receiving signals along with reducing the cost. We get high beamforming gain due to the analog domain and high multiplexing gain with reduced spectral efficiency due to the digital domain [12, 13]. Also, along with the cost reduction in mmWave systems, the complexity and the power efficiency of the system get improved because of using fewer RF chains compared to the antennas.

Furthermore, due to the above-mentioned propagation losses at the channel in mmWave systems, the response of the channel gets sparse because the propagation characteristics of mmWave are different compared to microwave systems [14, 15]. Also, due to the sparse channel in mmWave, the nature of the hybrid precoders gets sparse. Many attempts have been made in the literature to address the hybrid precoding problem using various algorithms [16]. In [17, 18], the authors propose a codebook-based hybrid precoding design by considering the sparse nature of the mmWave channel. Moreover, Orthogonal Matching Pursuit (OMP) is deemed a good candidate for designing hybrid precoders. However, with the increment in the number of transceivers, the computational complexity gets increased in OMP [19, 20]. As a result, it brings a poor estimation of the channel matrix due to the increment in the computational complexity, which reduces the spectral efficiency. Another approach, named as greedy Simultaneous Orthogonal Matching Pursuit (SOMP) technique, is used to reduce the system’s computational complexity. Although the computation complexity in terms of the number of iterations gets reduced in SOMP, the algorithm still leads to poor performance when we increase the dimension of transceiver chains [21] which reduces the spectral efficiency.

In [22], the authors propose a greedy frequency selective hybrid precoding algorithm based on Gram-Schmidt orthogonalization to reduce the complexity during the designing of precoders/combiners. The Gram-Schmidt orthogonalization algorithm is based on the greedy selection of columns of the transceiver matrix by considering minimal spatially active channel components to reduce the approximation error. In [23], the authors propose a frequency-selective hybrid precoding based on maximizing mutual information with the help of quantized codebooks. However, similar to the issues in OMP, the proposed schemes lead to poor performances when the number of transceiver elements gets increased.

In [24], the authors propose a Sparse Bayesian Learning (SBL) estimation scheme for the evaluation of posterior functions to the prior information, improving the channel’s estimation accuracy. Motivated by this research, the authors discuss the computation of sparse channels with the help of SBL estimation schemes [25–27]. They developed a Single Measurement Vector (SMV) for SBL-based mmWave MIMO channel estimation and then moved to the development of Multiple Measurement Vectors (MMV) for multi-user communication.

In [28], the authors propose a novel SBL framework to estimate the spatial signatures and temporal varying characteristics of the sparse virtual channel model. To avoid the unacceptable complexity, the expectation maximization (EM) algorithm is applied to approximate the maximum likelihood (ML) solution iteratively. Furthermore, in [29], the authors estimate the channel of a massive MIMO system by using the EM-based Variational Bayesian (EM-VB). By exploiting the channel reciprocity between the uplink and downlink, the downlink channel is estimated. In terms of methods, the authors use the SBL via EM approach for the computation of a time-varying channel in both of these papers.

In terms of precoder designing, the SBL method is used to minimize the error between the desired and the estimated precoders. For the estimation of missing information, the Expectation Maximization (EM) based SBL method is used during the designing of hybrid precoders, as EM-based SBL assists to compute the posterior distribution of hidden variables [30, 31]. In addition, it has been seen that the SBL works well for signal recovery, and in fact, it shows better performance compared to greedy methods and other basis pursuit methods. Despite exhibiting good performance, the dimension of the signal gets increased because we have to compute inverse elements having NxN size.

In [32], the authors propose a Generalized Approximate Message Passing (GAMP) algorithm for the estimation of the mmWave channel in a computationally efficient way. The integration of approximate message passing with EM assists to compute the distribution of true posterior approximately. Moreover, it has been seen that the performance of SBL with EM and GAMP is the same in terms of channel estimation accuracy, but the complexity is less in the latter one [33]. In this paper, we work on the design of hybrid precoders/combiners for mmWave massive MIMO hybrid architecture. The main purpose of using the hybrid architecture is to reduce the cost along with the power consumption, as RF chains are less than the transmitting antennas in the hybrid architecture. The nature of the precoders gets sparse in mmWave systems, we have proposed a deep learning-based architecture for designing the optimal hybrid precoders/combiners by getting motivated by the success of SBL via the GAMP algorithm for the estimation of mmWave channels in [34–36]. Firstly, the SBL via the GAMP algorithm is used for designing optimal hybrid precoders/combiners for mmWave massive MIMO systems in a computationally efficient way. To further improve the estimation accuracy and the channel overhead, we have proposed a deep learning-based approach, named Learned-SBL-GAMP (L-SBL-GAMP) algorithm, to design the hybrid precoders/combiners in mmWave massive MIMO systems. Actually, in the L-SBL-GAMP algorithm, all T layers are used to estimate the hybrid precoders/combiners and its matrices are sparse. The simulation results unveil the effectivities of the proposed deep learning-based method, where it can be seen that the proposed deep learning-based algorithm outperforms the existing methods (LS, OMP, SOMP, SBL-EM, and SBL-GAMP) by providing the reduced Normalized Mean Square Error (NMSE) value and the increased spectral efficiency.

In summary, Table 1 provides the pros/cons of the recent state-of-the-art work in the literature.

**Table 1:** A brief comparison of the pros/cons of the recent state-of-the-art work in the literature.

Survey Paper	Algorithm	Pros	Cons	Performance Comparison	Spectral Efficiency at SNR = 10dB	NMSE Value at SNR = 10dB	Accuracy in %
[17]	OMP	Computational Complexity is high $O(SN_{t}^{2}G+S^{3}G)$	OMP is a greedy approach whose performance is sensitive to the choice of the dictionary matrix and stopping criterion, which leads to convergence errors.	Better performance Compared to the LS	25 bits/s/Hz	-4	The improvement of Spectral Efficiency at SNR = 10dB for proposed L-SBL-GAMP is 41.2% compared to the LS, 26.5% is improved compared to OMP,11.7% is improved compared to SOMP,4.12% is improved compared to SBL-EM and 2.95% is improved compared to SBL-GAMP.
[18, 21]	SOME	High performance compared to OMP and Low Computational Complexity $O(N_{t}^{2}GN_{s})$	the SOMP is also sensitive to the choice of the dictionary matrix, which leads to convergence errors.	Better performance Compared to the OMP	30 bits/s/Hz	-9	
[31]	SBL-EM	Better Performance Compared to the OMP and SOMP	Computational Complexity is high *O*(*G*^3^) compared to the SBL-GMP	Better performance Compared to the OMP and SOMP	32.6 bits/s/Hz	-13.8	
[34]	SBL-GAMP	Computational Complexity is Low *O*(*N*_*t*_*G*) compared to SBL-EM	Same accuracy of SBL-EM	Better performance Compared to the OMP, SOMP and the same performance compared to SBL-EM	33 bits/s/Hz	-14	
Final Proposed Method	L-SBL-GAMP	High Accuracy		Better performance Compared to the existing algorithm.	34 bits/s/Hz	-15.4	

The main contributions and novelties of the paper are summarized as follows:

1. In this paper, we provide the mathematical modeling of optimal precoders/combiners for mmWave massive MIMO hybrid architecture.
2. To design hybrid precoders/combiners, we utilize the L-SBL via the GAMP algorithm, which reduces the computational complexity of the system compared to the EM-based SBL approach.
3. Due to the fixed nature of hyperparameters during the iterations in SBL via GAMP, the estimation accuracy of precoders gets reduced. Therefore, to tackle this issue, we propose a deep learning-based approach named learned SBL via GAMP.
4. In the proposed methodology, the hyperparameters get effectively tuned during each iteration, which assists in achieving the minimum loss function. The proposed DNN architecture can design the optimal hybrid precoders/combiners.
5. To validate the effectiveness of the proposed methodology, the experimental results are provided where it can be seen that the proposed methodology outperforms the existing approaches (LS, OMP, SOMP, SBL-EM, and SBL-GAMP) by providing less Normalized Mean Square Error (NMSE), losses, and the enhanced spectral efficiency.

### Notations

we opt for the following notations in this paper: **A**_(.*j*)_ and **A**_(*i*,*j*)_ represent the i^th^ row, j^th^ column, and (i, j)^th^ element of a matrix **A**, respectively. [⋅]* represents a conjugate operator, [⋅]^*H*^ represents a conjugate transpose operator, and [⋅]^*T*^ represents a transpose operator. **a** is a vector and *a* is a scalar. *E*(⋅) represents an expectation operator, ‖*X*‖_*F*_ represents a Frobenius norm of X, and denotes the l_2_ norm of X.

## System model

**Fig 1:**
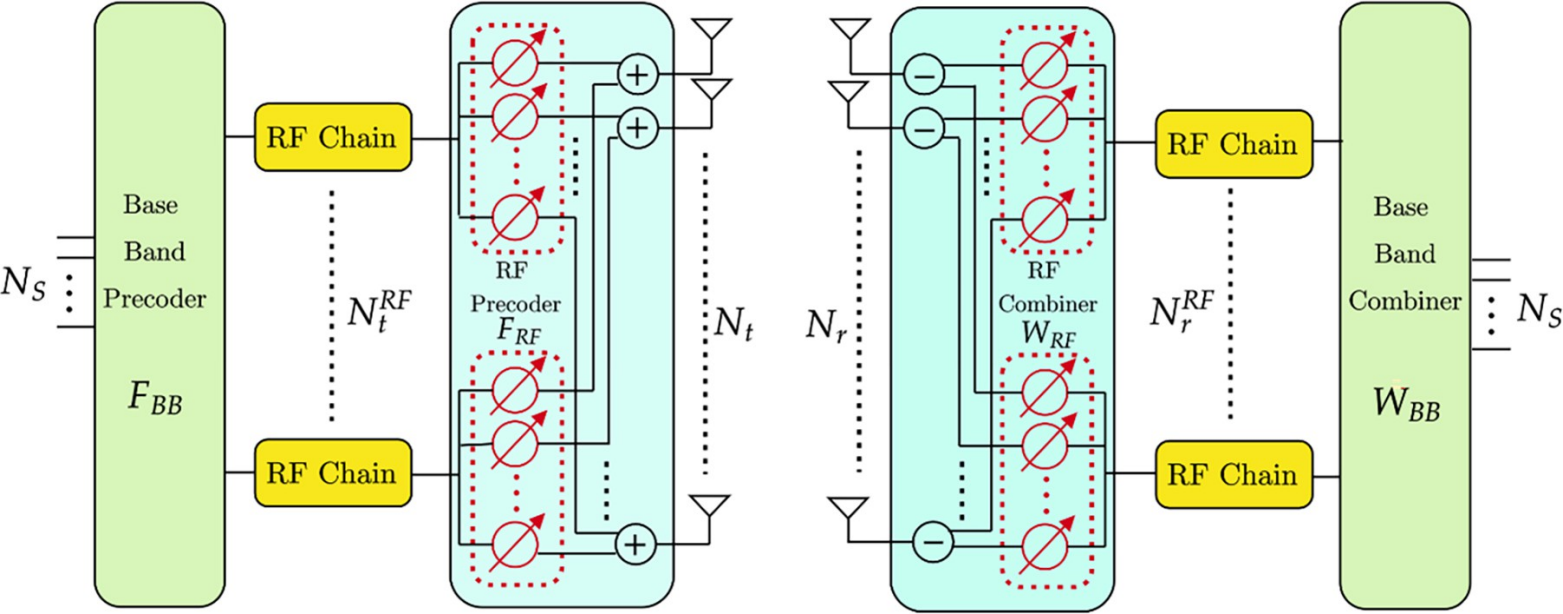
Hybrid mm wave MIMO architecture.

The architecture of hybrid precoders and combiners for the mmWave massive MIMO system is depicted in Fig 1. Let *N*_*t*_ be the transmitting elements at the transmission section, and several receiving antennas at the reception side. $N_{t}^{RF}$ represents the RF chains at the transmission, and represents the RF chains at the receiver side. It also *N*_*S*_ represents the number of data streams transmitted via RF chains at the transmitter and receiver sides. $F_{BB}\subset N_{t}^{RF}\times N_{S}$ denotes the baseband precoder, and $F_{RF}\subset N_{t}\times N_{r}^{RF}$ is the RF precoder at the transmitter side. $W_{BB}\subset N_{r}^{RF}\times N_{S}$ denotes the baseband combiner, and $W_{RF}\subset N_{r}\times N_{r}^{RF}$ represents the RF combiner. Furthermore, we impose total power constraints to normalize the baseband precoder **F**_*BB*_, so that ${\left\|F_{RF}F_{BB}\right\|}_{F}^{2}=N_{S}$ [31].

The numerical model for the channel with L paths is represented in Eq (1), which shows the channel correlation between the transmitting and receiving antennas. The geometric channel model is expressed as **H**⊂*N*_*r*_×*N*_*t*_, and it is written as:

$$H=\sum_{l=1}^{L}\alpha_{l}a_{r}(\phi_{r,l},\theta_{r,l})a_{t}^{H}(\phi_{t,l},\theta_{t,l})$$ (1)

Where represents the complex gain for the *l*th path. *φ*_*r*,*l*_,*θ*_*r*,*l*_ denote the Angles of Arrivals (AoAs) received for the *l*th path. Denote the Angles of Departures (AoDs) at the transmitting antenna for the *l*th path. **a**_*t*_(⋅) denotes the transmit array response and **a**_*r*_(⋅) denotes the receive array response. Due to mmWave frequencies, there are a small number of available channel paths *L*.

## Problem definition

This section deals with the designing of hybrid precoders/combiners that optimally increase the system’s achievable rate while suppressing channel training overhead. For the given system and channel models, the achievable rate can be written as [37]:

$$R=\log_{2}|I+R_{n}^{-1}W^{H}HFF^{H}H^{H}W|$$ (2)

Where **F** = **F**_*RF*_**F**_*BB*_ denotes the analog/digital precoder, and denotes the analog/digital combiners. The covariance matrix can be written as:

$$R_{n}^{}=\frac{1}{SNR}W^{H}W$$ (3)

The signal-to-noise ratio (*SNR*) of the system can be written as:

$$SNR=\frac{P_{T}}{N_{S}\sigma_{n}^{2}}W^{H}W$$ (4)

Where represents the total transmit power, and denotes the noise power.

The designing of optimal hybrid precoders and combiners is required to improvise mutual information between the baseband precoder and RF precoder, which can be written as [31]:

$$\left(F_{BB}^{opt},F_{RF}^{opt})=\arg\underset{F_{BB},F_{RF}}{\max}I(F_{BB},F_{RF}\right)$$ (5)

$$\left(W_{BB}^{opt},W_{RF}^{opt})=\arg\underset{W_{BB},W_{RF}}{\max}I(W_{BB},W_{RF}\right)$$ (6)

The direct maximization process is difficult; therefore, an ideal digital precoder and combiner method is used. The ideal digital precoder and combiner can be written as:

$$F_{opt}=V(:,1:N_{S})\in C^{N_{t}\times N_{S}}$$ (7)

$$W_{opt}=U(:,1:N_{S})\in C^{N_{r}\times N_{S}}$$ (8)

Where denotes the right singular vector matrices, and $U\in C^{N_{r}\times N_{r}}$ denotes the left singular vector matrices. The values of matrices are estimated from the Singular Value Decomposition (SVD) **H**.

The problem of maximizing the mutual information concerning the precoders **F**_*BB*_ and **F**_*RF*_ can be well approximated by the sparse matrix reconstruction problem, and it is formulated as below:

The equivalent precoders and combiners design problem for the mmWave MIMO system to achieve the best approximation to the ideal precoder **F**_*opt*_ and ideal combiner **W**_*opt*_ is shown below in Eqs (9) and (11) [31]:

$${\hat{F}}_{BB}^{opt}=\arg\;\underset{{\hat{F}}_{BB}}{\min}{\left\|F_{opt}-A_{T}{\hat{F}}_{BB}\right\|}_{F}$$ (9)

$$s.t{\left\|diag({\hat{F}}_{BB}{\hat{F}}_{BB}^{H})\right\|}_{0}\leq K,\|A_{T}{\hat{F}}_{BB}\|=N_{S}$$ (10)

Where $A_{T}≜[a_{t}(\theta_{t,l}),a_{t}(\theta_{t,2}),\ldots .,a_{t}(\theta_{t,G})]\in C^{N_{t}\times G}$ denotes the dictionary matrix of transmit array response, and $A_{R}≜[a_{r}(\theta_{r,l}),a_{t}(\theta_{r,2}),\ldots .,a_{r}(\theta_{r,G})]\in C^{N_{r}\times G}$ denotes the dictionary matrix of receive array response.

The first constraint arises because ${\hat{F}}_{BB}^{opt}\in C^{G\times N_{S}}$ can have only $N_{t}^{RF}\leq K$ nonzero rows corresponding to the number of active RF chains, while the second constraint $\|A_{T}{\hat{F}}_{BB}\|=N_{S}$.


$${\hat{W}}_{BB}^{opt}=\arg\;\underset{{\hat{W}}_{BB}}{\min}{\left\|R_{yy}^{1/2}(W_{MMSE}-A_{R}{\hat{W}}_{BB})\right\|}_{F}$$ (11)

$$s.t{\left\|diag({\hat{W}}_{BB}{\hat{W}}_{BB}^{H})\right\|}_{0}\leq K,\|W_{RF}{\hat{W}}_{BB}\|=N_{S}$$ (12)

## Sparse Bayesian learning (SBL) algorithm

In this section, a two-layer hierarchical prior model is proposed to calculate the sparsity of a Bayesian learning model. The first layer represents the Gaussian prior distribution [32]:

$$p(F_{BB}|\alpha )=\prod_{n=1}^{G}p(F_{BB_{n}}|\alpha_{n})=\prod_{n=1}^{G}p(F_{BB_{n}}|0,\alpha_{n}^{-1})$$ (13)

Where denotes the non-negative hyperparameter, which controls the sparsity coefficient $F_{BB_{n}}$. In the next layer, hyperpriors are computed with the help of Gamma distributions:

$$p(\alpha )=\prod_{n=1}^{G}Gamma(\alpha_{n}|a,b)=\prod_{n=1}^{G}\Gamma^{-1}(a)b^{a}\alpha_{n}^{a-1}e^{-b\alpha_{n}}$$ (14)

$$\Gamma (a)=\int_{0}^{\infty }t^{a-1}e^{-t}dt$$ (15)

$$p(\gamma )=Gamma(\gamma |c,d)=\Gamma {\left(c\right)}^{-1}d^{c}\gamma^{c-1}e^{-d\gamma }$$ (16)

The sparse signal and the hyperparameters are learned using the Expectation-Maximization (EM) algorithm. In the EM algorithm, **F**_*BB*_ is a hidden variable. Also, using the iterative method, we strengthen a lower bound value on probability *p*(**α**,*γ*|**F**_*opt*_). The EM algorithm works in two steps named E-step and M-Step, respectively. Firstly, the **F**_*BB*_ is computed, and it is given as:

$$p(F_{BB}|F_{opt},\alpha^{\left(t\right)},\gamma^{\left(t\right)})\propto p(F_{BB}|\alpha^{\left(t\right)})p(F_{opt}|F_{BB},\gamma^{\left(t\right)})$$ (17)

From the posterior function $p(F_{BB}|F_{opt},\alpha^{\left(t\right)},\gamma^{\left(t\right)})$, the Gaussian distribution of its mean and covariance matrix is written as:

$$\mu =\gamma^{\left(t\right)}{\mathrm{\Phi A}}_{T}^{T}F_{opt}$$ (18)

$$\Phi ={\left(\gamma^{\left(t\right)}A_{T}^{T}A_{T}+D\right)}^{-1}$$ (19)

The posterior $p(F_{BB}|F_{opt},\alpha^{\left(t\right)},\gamma^{\left(t\right)})$ obeys the Gaussian distribution, and its parameters are:

$$D=diag(\alpha_{1}^{t},\ldots ..,\alpha_{N}^{t})$$ (20)

$$E_{{}_{F_{BB}|F_{opt},}}{}_{\alpha^{\left(t\right)},\gamma^{\left(t\right)}}\left[\log p(\alpha ,\gamma |F_{opt}\right]$$ (21)

$$p(F_{BB}|F_{opt},\alpha^{\left(t\right)},\gamma^{\left(t\right)})$$ (22)

The Q-function is maximized in M-steps by keeping {**α**,*γ*}, which leads to the following update rules:

$$\alpha_{n}^{\left(t+1\right)}=\frac{2a-1}{\langle F_{BB}{}_{n}^{2}\rangle +2b}$$ (23)

$$\gamma^{\left(t+1\right)}=\frac{M+2c-2}{\langle {\left\|F_{opt}-A_{T}F_{BB}\right\|}_{2}^{2}\rangle +2d}$$ (24)

Where is the expectation concerning? It is observed that the EM algorithm for every iteration has to update the posterior distribution with *G*×*G* computation inverse. Hence, the EM-based sparse Bayesian has complexity in the range of *O*(*G*^3^) flops, which increases further concerning larger dimensions [32].

The optimal hybrid precoder and combiner are designed with the help of SBL based EM method. The SBL-EM method achieves high spectral efficiency and it increases the training overhead with computational complexity. To minimize the number of iterations and complexity, we use the SBL via the GAMP algorithm. The SBL via the GAMP algorithm does not require an inverse operation. It only needs a simple multiplication operation.

## Proposed learned SBL-GAMP architecture

In this section, we first discuss the SBL via the GAMP algorithm and then move to the proposed L-SBL-GAMP algorithm.

### SBL-GAMP algorithm

The GAMP algorithm attains the target with a smaller number of iterations and training overhead [32]. The Bayesian iterative technique, named GAMP, is adapted to estimate the optimal hybrid precoder of mmWave systems. The proposed algorithm provides low computational complexity and finds the estimation of a posterior distribution conditioned on the prior distribution **F**_*BB*_. In the EM algorithm, the posterior estimate distribution **F**_*BB*_ is used to swap the true posterior distribution. Moreover, the hyperparameters are considered as known and fixed in the GAMP algorithm. Depending on the estimated values **F**_*BB*_, its parametric values are updated in the M-step.

In the GAMP algorithm, we use the central-limit-theorem to approximate variable and factor nodes, which enhances the performance of the system. The SBL-GAMP framework adopts two important approximation methods. In the first approximation method, the GAMP assumes posterior independence among hidden variables {**F**_*BBn*_}, which *θ* = {**α**,*γ*} denotes the hyperparameters.

The true posterior distribution approximates as *p*(**F**_*BBn*_|**F**_*opt*_,**θ**), and it is computed as:

$$\hat{p}(F_{BB}{}_{n}|F_{opt},{\hat{r}}_{n},\tau_{n}^{r},\theta )=\frac{p(F_{BB_{n}}|\theta )Ν(F_{BB_{n}}|{\hat{r}}_{n},\tau_{n}^{r})}{\int_{F_{BB}}p(F_{BB_{n}}|\theta )Ν(F_{BB_{n}}|{\hat{r}}_{n},\tau_{n}^{r})}$$ (25)

Let ${\hat{r}}_{n}$ and are different variables that are updated for every process value of the GAMP algorithm. With the help of Gaussian Prior Distribution, the approximate posterior $\hat{p}(F_{BB}{}_{n}|F_{opt},{\hat{r}}_{n},\tau_{n}^{r},\theta )$ of mean and variance is given in Eqs (26) and (27).


$$\mu_{n}^{F_{BB}}=\frac{{\hat{r}}_{n}}{1+\alpha_{n}\tau_{n}^{r}}$$ (26)

$$\phi_{n}^{F_{BB}}=\frac{\tau_{n}^{r}}{1+\alpha_{n}\tau_{n}^{r}}$$ (27)

$F_{m}=a_{m}^{T}F_{BB}$ denotes the noiseless output of the other approximation method, where $a_{m}^{T}$ specifies the m^th^ row in **A**_*T*_. The GAMP equates to the exact marginal posterior *p*(*F*_*m*_|**F**_*optm*_,*θ*):

$$\hat{p}(F_{m}|F_{opt},{\hat{p}}_{m},\tau_{m}^{p},\theta )=\frac{p(F_{opt}{}_{m}|F_{m},\theta )N(F_{m}|{\hat{p}}_{m},\tau_{m}^{p})}{\int_{F}p(F_{opt}{}_{m}|F_{m},\theta )N(F_{m}|{\hat{p}}_{m},\tau_{m}^{p})}$$ (28)

Where ${\hat{p}}_{m}$ and $\tau_{m}^{p}$ are the parameters that are iteratively updated during the iterative process of GAMP.

Using the Gaussian prior distribution $\hat{p}(F_{m}|F_{opt}{}_{m},{\hat{p}}_{m},\tau_{m}^{p},\theta )$ defines the mean and variance, and it is given in Eqs (29) and (30).


$$\mu_{m}^{F}=\frac{\tau_{m}^{p}\gamma F_{opt}{}_{m}+{\hat{p}}_{m}}{1+\gamma \tau_{m}^{p}}$$ (29)

$$\phi_{m}^{F}=\frac{\tau_{m}^{p}}{1+\gamma \tau_{m}^{p}}$$ (30)

With the above two approximation methods, the two following important scalar functions are defined as *gin*(⋅) and *gout*(⋅), which are utilized for the proposed approach. *gin*(⋅) denotes the scalar function of input, which defines the posterior mean $\mu_{n}^{F_{BB}}$, and it is expressed by using Minimum Mean Squared Error (MMSE) mode [34]:

$$gin({\hat{r}}_{n},\tau_{n}^{r},\theta )=\mu_{n}^{F_{BB}}=\frac{{\hat{r}}_{n}}{1+\alpha_{n}\tau_{n}^{r}}$$ (31)

To obtain the estimated variance $\phi_{n}^{F_{BB}}$, we take the partial derivative $\tau_{n}^{r}$ $gin({\hat{r}}_{n},\tau_{n}^{r},\theta )$ with respect to $\tau_{n}^{r}$:

$$\tau_{n}^{r}\frac{\partial }{\partial {\hat{r}}_{n}}gin({\hat{r}}_{n},\tau_{n}^{r},\theta )=\varphi_{n}^{F_{BB}}=\frac{\tau_{n}^{r}}{1+\alpha_{n}\tau_{n}^{r}}$$ (32)

Moreover, *gout*(⋅) denotes the output scalar function, and its posterior mean $\mu_{m}^{F}$ is defined as:

$$gout({\hat{p}}_{m},\tau_{m}^{p},\theta )=\frac{1}{\tau_{m}^{p}}(\mu_{m}^{F}-{\hat{p}}_{m})=\frac{1}{\tau_{m}^{p}}(\frac{\tau_{m}^{p}\gamma F_{opt}{}_{m}+{\hat{p}}_{m}}{1+\gamma \tau_{m}^{p}}-{\hat{p}}_{m})$$ (33)

Taking the partial derivative of $gout({\hat{p}}_{m},\tau_{m}^{p},\theta )$ to obtain the posterior variance $\phi_{m}^{F}$:

$$\tau_{m}^{p}\frac{\partial }{\partial }gout({\hat{p}}_{m},\tau_{m}^{p},\theta )=\frac{\phi_{m}^{F}-\tau_{m}^{p}}{\tau_{m}^{p}}=\frac{-\gamma \tau_{m}^{p}}{1+\gamma \tau_{m}^{p}}$$ (34)

The GAMP algorithm finds the posterior mean $\mu_{n}^{F_{BB}}(k)$ and variance **F**_*BBn*_ at the k iteration for the given input *gin*(⋅) and output *gout*(⋅) scalar functions. Finally, the approximate posterior distributions for the variables **F**_*BB*_ **F**_*m*_ = **A**_*T*_**F**_*BB*_ are derived by using the proposed GAMP algorithm. The GAMP algorithm does not require an inverse operation. It only needs a simple multiplication operation, which can be scaled as *O*(*N*_*t*_*G*). Thus, its computational complexity gets considerably reduced.

As far as learning hyperparameters is concerned, the EM method is used to estimate hyperparameters by considering hidden variables **F**_*BB*_ and the iteratively improving Q-function:

$$\theta^{\left(t+1\right)}=\arg\;\underset{\theta }{\max}Q(\theta |\theta^{\left(t\right)})=E_{{}_{F_{BB}|F_{opt},}\theta (t)}[\log p(\theta |F_{BB},F_{opt})]$$ (35)

To find the number of M-steps for the analysis of hyperparameters {*α*_*n*_}, we take the first-order derivative of the Q-function for *α*_*n*_:

$$\frac{\partial }{\partial \alpha_{n}}Q(\theta |\theta^{\left(t\right)})=\frac{\partial }{\partial \alpha_{n}}E_{{}_{F_{BB}|F_{opt},}\theta (t)}[\log p(\theta |F_{BB},F_{opt})]$$ (36)

$$=\frac{\partial }{\partial \alpha_{n}}E_{{}_{F_{BB}|F_{opt},}\theta (t)}[\log p(\theta |F_{BB},F_{opt})p(\alpha_{n};a,b)]$$ (37)

$$=\frac{1}{2\alpha_{n}}-\frac{\langle F_{BB}{}_{n}^{2}\rangle }{2}+\frac{-1}{\alpha_{n}}-b$$ (38)

Where represents the expectation operator regarding *p*(**F**_*BB*_|**F**_*opt*_,**θ**^(*t*)^). The approximate posterior distribution **F**_*BBn*_ is used due to the unavailability of a true posterior. The GAMP algorithm is used to approximate the posterior distribution by replacing the true posterior distribution. From the Gaussian distribution process, the approximate posterior distribution $\hat{p}(F_{BB}{}_{n}|F_{opt},{\hat{r}}_{n}(k_{0}),\tau_{n}^{r}(k_{0}),\theta^{\left(t\right)})$ defines the mean and variance, which are given in Eqs (26) and (27)

$$\langle F_{BB}{}_{n}^{2}\rangle =\frac{{\left({\hat{r}}_{n}(k_{0})\right)}^{2}}{{\left(1+\alpha_{n}^{\left(t\right)}\tau_{n}^{r}(k_{0})\right)}^{2}}+\frac{\left(\tau_{n}^{r}(k_{0})\right)}{\left(1+\alpha_{n}^{\left(t\right)}\tau_{n}^{r}(k_{0})\right)}$$ (39)

$$\alpha_{n}^{\left(t+1\right)}=\frac{2a-1}{2b+\langle F_{BB}{}_{n}^{2}\rangle }\;\forall n\in \{1,\ldots .,N_{t}^{RF}\}$$ (40)

Here, hyperparameters are required to be updated. The function of a hyperparameter is to inverse noise variance. The approximate posterior distribution of output without any disturbance is considered in the GAMP approach. The *F* is then assigned to the hidden variables for understanding noise variance, and it is given as:

$$\gamma^{\left(t+1\right)}=\arg\;\underset{\gamma }{\max}E_{{}_{F|F_{opt},}\theta (t)}[\log p(F_{opt}|F,\gamma )p(\gamma ;c,d)]$$ (41)

From the partial derivative of Q-function regarding *γ* projects:

$$\frac{\partial }{\partial \gamma }E_{{}_{F|F_{opt},}\theta (t)}[\log p(F_{opt}|F_{BB},\gamma )p(\gamma ;c,d)]$$ (42)

$$=\frac{M}{2\gamma }-\frac{1}{2}\sum_{m=1}^{M}\langle {\left(F_{opt}{}_{m}-F_{m}\right)}^{2}\rangle +\frac{c-1}{\gamma }-d$$ (43)

〈⋅〉 specifies the expectation for $p(F_{m}|y,{\hat{p}}_{m}(k_{0}),\tau_{m}^{p}(k_{0}),\theta^{\left(t\right)})$, and the approximate posterior distribution *F*_*m*_. We know that *F*_*m*_ tracks the Gaussian distribution in terms of mean and variance, and it is expressed as:

$$\langle {\left(F_{opt}{}_{m}-F_{m}\right)}^{2}\rangle ={\left(F_{opt}{}_{m}-\mu_{m}^{F}\right)}^{2}+\phi_{m}^{F}$$ (44)

where $\mu_{m}^{F}$ and $\phi_{m}^{F}$ from (29)–(30) with $\left\{{\hat{p}}_{m},\tau_{m}^{p}\right\}$ relocated with $\left\{{\hat{p}}_{m}(k_{0}),\tau_{m}^{p}(k_{0})\right\}$ and *γ* is changed with *γ*^(*t*)^. By equating the derivative to zero, the updated rule for:

$$\gamma^{\left(t+1\right)}=\frac{M+2c-2}{2d+\sum_{m}\langle {\left(F_{opt}{}_{m}-F_{m}\right)}^{2}\rangle }$$ (45)

As of now, the GAMP-based sparse Bayesian learning algorithm is derived. For a better understanding, we have summarized the SBL-GAMP approach in algorithm 1 [32].

**Algorithm 1: SBL-GAMP**

**Initialization:** For known **θ**^(*t*)^; assign $k=0,{\hat{s}}_{m}^{\left(-1\right)}=0,\forall m\in \{1,\ldots ,M\};{\left\{\mu_{n}^{F_{BB}}(k)\right\}}_{n=1}^{G}$ and ${\left\{\varphi_{n}^{F_{BB}}(k)\right\}}_{n=1}^{G}$ are initialized for prior distribution with mean and variance. Continue the process until ${\sum_{n}\left|\mu_{n}^{F_{BB}}(k+1)-\mu_{n}^{F_{BB}}(k)\right|}^{2}\leq \varepsilon$ Let *ε* be pre-specified error tolerance.

**Initialization:** given and *γ*^(0)^

$$F_{opt}{}_{m}(k)=\sum_{n}a_{mn}\mu_{n}^{F_{BB}}(k)$$

$$\tau_{m}^{p}(k)=\sum_{n}a_{mn}^{2}\phi_{n}^{F_{BB}}(k)$$

$${\hat{p}}_{m}(k)=F_{opt}{}_{m}(k)-\tau_{m}^{p}(k){\hat{s}}_{m}(k-1)$$

$${\hat{s}}_{m}(k)=gout({\hat{p}}_{m}(k),\tau_{m}^{p}(k),\theta^{\left(t\right)})$$

$$\tau_{m}^{s}(k)=\frac{\partial }{\partial {\hat{p}}_{m}}gout({\hat{p}}_{m}(k),\tau_{m}^{p}(k),\theta^{\left(t\right)})$$

$$\tau_{n}^{s}(k)={\left(\sum_{m}a_{mn}\tau_{m}^{s}(k)\right)}^{-1}$$

$${\hat{r}}_{n}(k)=\mu_{n}^{F_{BB}}(k)+\tau_{n}^{s}(k)\sum_{m}a_{mn}{\hat{s}}_{m}(k)$$

$$\mu_{n}^{F_{BB}}(k+1)=gin({\hat{r}}_{n}(k),\tau_{n}^{r}(k),\theta^{\left(t\right)})$$

$$\varphi_{n}^{F_{BB}}(k+1)=\tau_{n}^{r}\frac{\partial }{\partial {\hat{r}}_{n}}gin({\hat{r}}_{n}(k),\tau_{n}^{r}(k),\theta^{\left(t\right)})$$

Based on derivates of the GAMP algorithm, hyperparameters are updated according to (40) and (45) using the EM method.

$$\alpha_{n}^{\left(t+1\right)}=\frac{2a-1}{2b+\langle F_{BB}{}_{n}^{2}\rangle }\;\forall n\in \{1,\ldots .,N\}$$

$$\gamma^{\left(t+1\right)}=\frac{M+2c-2}{2d+\sum_{m}\langle {\left(F_{opt_{m}}-F_{m}\right)}^{2}\rangle }$$

$$\mathrm{Output}-F_{BB_{T}}=\mu_{n}^{F_{BB}}(k+1)$$

However, in SBL via GAMP, the hyperparameter is considered fixed for all the iterations, which reduces the estimation accuracy of precoders. To overcome this issue, the learned SBL via GAMP is proposed. In the learned SBL via GAMP, for each layer, the hyperparameters get changed and tuned to obtain the minimum loss function.

### Learned SBL-GAMP architecture

**Fig 2:**
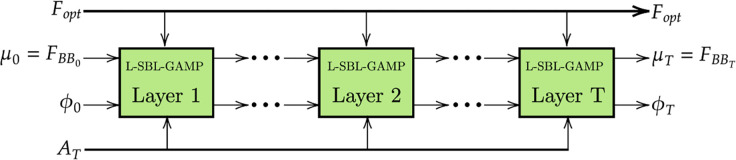
L-SBL-GAMP architecture.

The architecture of L-SBL-GAMP with multiple layers is shown in Fig 2. Each layer of the L-SBL-GAMP is similar to an iteration of the SBL-GAMP algorithm. The inputs of the L-SBL-GAMP network are the **F**_*opt*_ (Ideal digital Precoder) and **A**_*T*_ (transmit array response). The outputs of the L-SBL-GAMP network are baseband precoder and trainable parameters **θ** = {**W**} = {**α**,*γ*}. In algorithm 2, for the first layer, fix them to learn **W**_1_ = {**α**_1_,*γ*_1_} and re-learn **W**_1_. In layer 2, fix the to learn them **W**_2_ = {**α**_2_,*γ*_2_} and re-learn **W**_0_, **W**_1_ and **W**_2_. Similarly, the process will continue till the T layer, and it is shown in algorithm 2.

**Algorithm 2: Learned SBL-GAMP**

Initialization: Input: Training set $\left\{F_{opt}^{i},A_{T}^{i}\},\theta_{0}=\{W_{0}\}=\{\alpha_{0},\gamma_{0}\right\}$ For *t* = 1,…,*T* **do**

Initialize: **W**_*t*_ = **W**_*t*−1_

for R steps do

Learn **W**_*t*_ = {**α**_*t*_,*γ*_*t*_} with fixed $\theta_{t-1}={\left\{W_{l}\right\}}_{l=1}^{t-1}$ to minimize the loss function

First estimate optimal precoder

${\hat{F}}_{BB}^{i}=G(F_{opt}^{i},F_{RF}^{i};\theta )$ and then find the loss function

$\frac{1}{m}{\sum_{i=1}^{m}\left\|F_{opt}^{i}-A_{T}^{i}{\hat{F}}_{BB}^{i}\right\|}_{2}^{2}$ **End for**

for R steps do

Re-Learn $\theta_{t}={\left\{W_{l}\right\}}_{l=1}^{t}$ to minimize the loss function

Estimate optimal precoder

${\hat{F}}_{BB}^{i}=G(F_{opt}^{i},A_{T}^{i};\theta )$ and then calculate the loss function

$\frac{1}{m}{\sum_{i=1}^{m}\left\|F_{opt}^{i}-A_{T}^{i}{\hat{F}}_{BB}^{i}\right\|}_{2}^{2}$ **End for**

**End for**

Output: **θ**_*T*−1_

Algorithm 2, **θ** = {**W**} = {**α**,*γ*} denotes the set of learnable parameters in the t-th layer. The total number of L-SBL-GAMP layers is denoted as T, and R denotes the total number of mini-batches used in the training of the L-SBL-GAMP layer. The output vector $F_{opt}^{i}$ is related to the sparse baseband precoder vector $F_{BB}^{i}$:

$$F_{opt}^{i}=A_{T}^{i}F_{BB}^{i}$$ (46)

The loss function used in the training of the L-SBL-GAMP layer is the mean square error between the true sparse vector and the current training layer output. The expression for the mean square error loss function is given by:

$$L=\frac{1}{m}{\sum_{i=1}^{m}\left\|F_{opt}^{i}-A_{T}^{i}{\hat{F}}_{BB}^{i}\right\|}_{2}^{2}$$ (47)

where m denotes the number of training samples in a mini-batch and **G** represents the function learned by L-SBL-GAMP. A layer-by-layer method is used, and the Adam optimizer is used in the back-propagation process to optimize the final value. The L-SBL-GAMP network is implemented in Python using the neural network libraries Keras and TensorFlow.

After estimating the sparse precoder ${\hat{F}}_{BB}\in C^{G\times N_{S}}$, we are following a two-step procedure to extract the baseband precoder $F_{BB}\in C^{N_{t}^{RF}\times N_{S}}$. We calculate the transmit baseband precoder matrix from ${\hat{F}}_{BB}$. Firstly, we separate the G − K rows ${\hat{F}}_{BB}$ to get ${\hat{F}}_{BB}\in C^{K\times N_{S}}$. The rows of the matrix are then removed to finally obtain the matrix. The transmit RF precoder **F**_*RF*_ can be extracted by keeping the $N_{t}^{RF}$ columns corresponding to the rows extracted from ${\hat{F}}_{BB}$.

The above same procedure is utilized to calculate the baseband combiner ${\hat{W}}_{BB}$ and **W**_*RF*_ RF combiner. They can be extracted from by retaining the $N_{r}^{RF}$ columns corresponding to the rows extracted from ${\hat{W}}_{BB}$.

Moreover, and is the optimum MMSE combiner.

The estimated baseband precoder, RF precoder, baseband combiner, and RF combiner are substituted in Eq (2) to obtain the achievable rate or Spectral Efficiency [37].

Finally, the Normalized Mean Square Error (NMSE) is used to evaluate the performance of the L-SBL-GAMP network:

$$\mathrm{NMSE}=\frac{E\{{\left\|F_{opt}-F_{RF}{\hat{F}}_{BB}\right\|}_{2}^{2}\}}{E\{{\left\|F_{opt}\right\|}_{2}^{2}\}}$$ (48)

### Computational complexity and performance analysis

Sparse Bayesian Learning (SBL) is a popular method for sparse signal recovery or optimal hybrid precoder design. SBL-EM (Expectation-Maximization) and SBL-GAMP (Gaussian Approximation Message Passing) are two variants of SBL that differ in their algorithms for estimating the sparse signal or designing the hybrid precoder.

In terms of performance, the accuracy of the optimal hybrid precoder design using SBL-EM is almost the same compared to SBL-GAMP, but at the cost of higher computational complexity.

The L-SBL-GAMP algorithm is an extension of the SBL- GAMP algorithm that incorporates a deep neural network to improve performance. The L-SBL-GAMP approach can achieve high accuracy while reducing the computational complexity compared to SBL-EM. However, the performance of the L-SBL-GAMP approach may depend on the quality and quantity of training data used to train the neural network.

Overall, while the L-GAMP-SBL algorithm has a higher computational complexity than GAMP-SBL and reduces computational complexity than SBL-EM, the improved performance it provides may make it the best choice in many practical applications where accuracy is critical. The choice of algorithm ultimately depends on the specific requirements of the application and the trade-off between computational complexity and performance.

SBL-EM has a computational complexity of *O*(*G*^3^), where G is the number of grid matrices. The SBL-GAMP algorithm does not require an inverse operation. It only needs a simple multiplication operation, which can be scaled as *O*(*N*_*t*_*G*). Thus, its computational complexity gets considerably reduced.

The computational complexity of L-SBL- GAMP is dominated by the training process, which typically has a complexity of *O*(ETN_t_G), where E is the number of epochs, T is the number of training samples or layers, N_t_ is the number of transmit antenna and G is the number of grid matrix or dictionary matrix.

In general, the computational complexity of L-SBL- GAMP is higher than that of SBL- GAMP, due to the added complexity of the training process. However, this added complexity may be justified if the improved accuracy of L-SBL-GAMP leads to better performance in practice. It is also worth noting that there are techniques for reducing the computational complexity of the training process, such as using mini-batch training or pre-training the neural network on a smaller dataset before fine-tuning it on the full dataset.

The L-SBL- GAMP algorithm has a higher computational complexity than SBL- GAMP, as it involves additional processing steps required to train and use the deep neural network. However, the computational complexity of L-SBL-GAMP is still significantly lower than that of SBL-EM.

Overall, while the L-SBL- GAMP algorithm has a higher computational complexity than SBL-GAMP, it is still significantly more efficient than SBL-EM, and the improved performance it provides may make it the best choice in many practical applications.

## Results

In this section, the proposed L-SBL-GAMP algorithm is used to estimate hybrid precoders/combiners in the hybrid architecture. We assume 64 antennas are employed both at the transmitting and receiving ends, and 4 RF chains are considered. The channel matrix dataset is generated. A random noise is added to the communication link, as it happens in the conventional communication system. A data point in the dataset is nothing, but a pair of noisy channel and codebook indices. This created dataset is used to train the proposed deep learning neural network, which is utilized to find the loss function. Our model is implemented using the Keras libraries [36] with a Theano backend. The Adam optimizer is used with a momentum of 0.5, a batch size of 512, and a 0.005 learning rate is used. The performance of the proposed L-SBL-GAMP algorithm is compared with the existing algorithms, such as OMP, SOMP, and SBL-EM. The following parameters are used to simulate the proposed and the existing algorithms.

Let the transmitting and receiving elements are considered as *N* = *N*_*r*_ = *N*_*t*_ = 64, where the count of RF chains is considered as $N_{t}^{RF}=N_{r}^{RF}=4$, and several data streams are assumed for the simulation.

**Fig 3:**
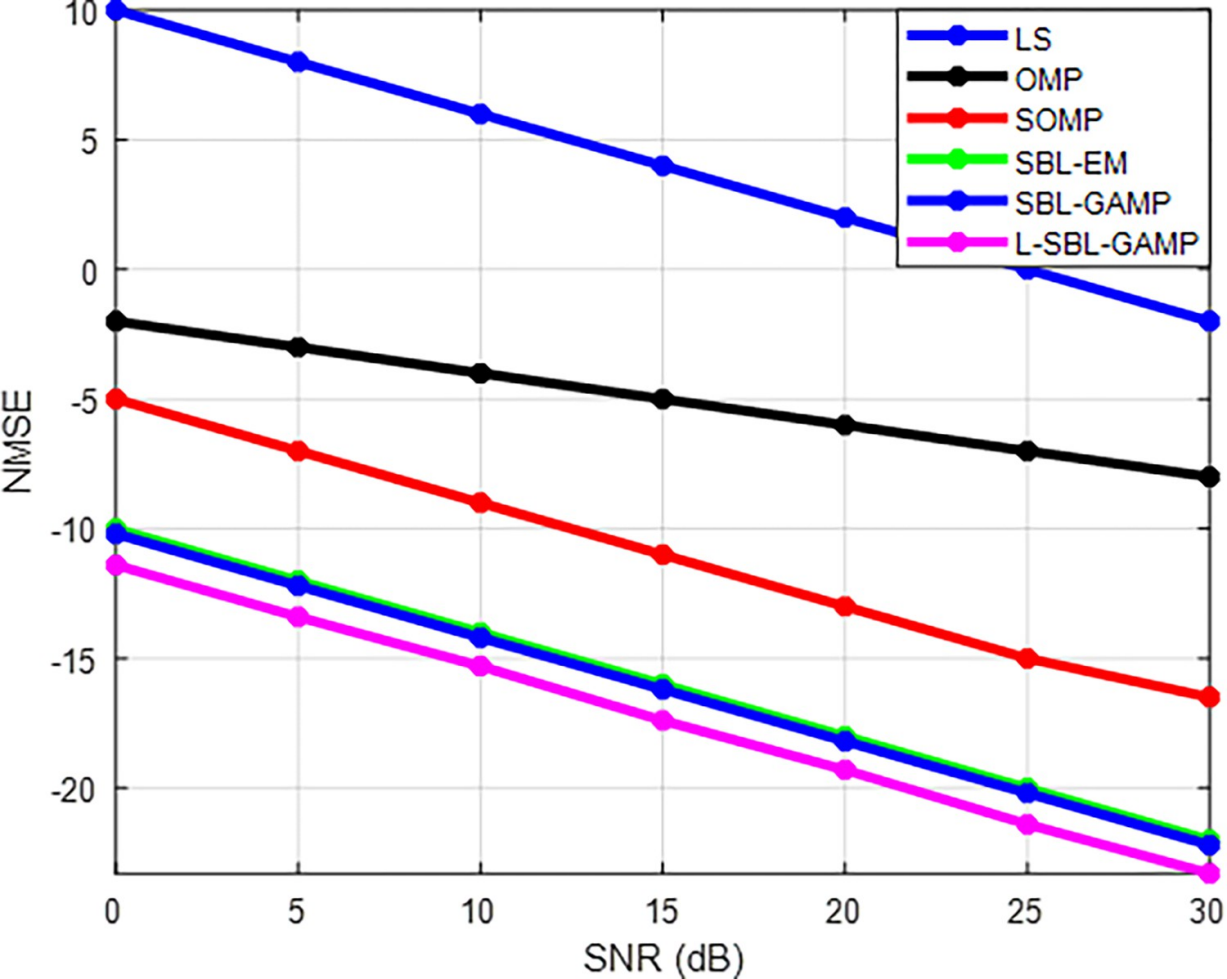
NMSE Vs SNR (dB).

Fig 3 shows NMSE vs. SNR for the proposed and existing algorithms. The proposed L-SBL-GAMP algorithm provides better results compared to the OMP, SOMP, SBL-EM, and SBL-GAMP. In Fig 3, the accuracy of the optimal hybrid precoder design using SBL-EM is almost the same compared to the SBL-GAMP method, but computational complexity is high in SBL-EM compared to the SBL-GAMP. The proposed L-SBL-GAMP algorithm is an extension of the SBL- GAMP algorithm that incorporates a deep neural network to improve performance. The L-SBL-GAMP approach can achieve high accuracy while reducing the computational complexity compared to SBL-EM. Overall, the L-GAMP-SBL algorithm has a higher computational complexity than GAMP-SBL and lower computational complexity than the SBL-EM method. The choice of algorithm ultimately depends on the specific requirements of the application and the trade-off between computational complexity and performance.

**Fig 4:**
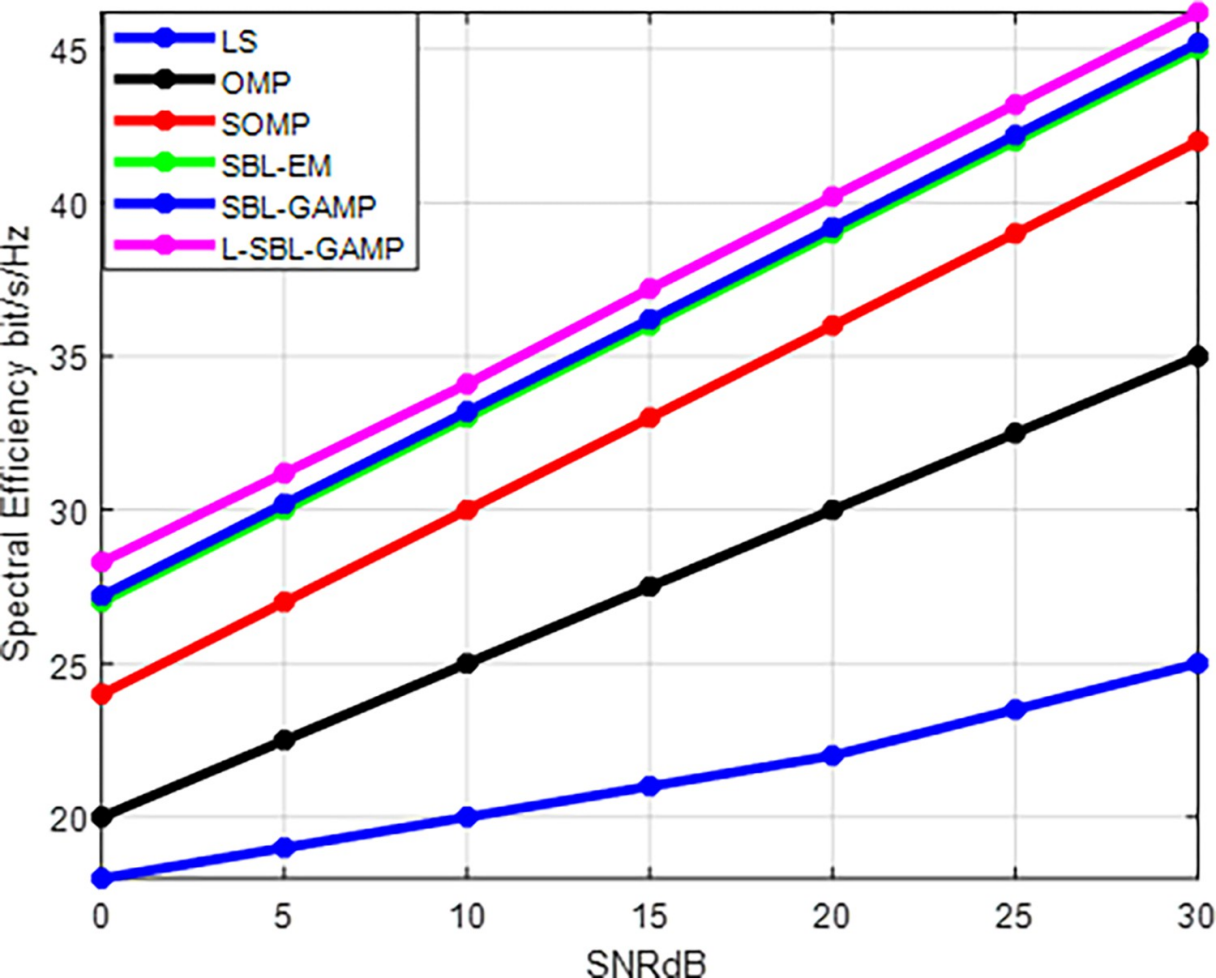
SNR (dB) Vs spectral efficiency (bits/s/Hz).

The same parameters are used for both Figs 3 and 4. The spectral efficiency against SNR for the proposed L-SBL-GAMP and existing algorithms is shown in Fig 4. The proposed L-SBL-GAMP algorithm achieves better spectral efficiency with a small number of iterations compared to the other existing algorithms. The SBL-GAMP algorithm and SBL-EM method achieve approximately the same achievable rates, but the computational complexity is higher in the SBL-EM method compared to the SBL-GAMP and proposed L-SBL-GAMP algorithm.

**Fig 5:**
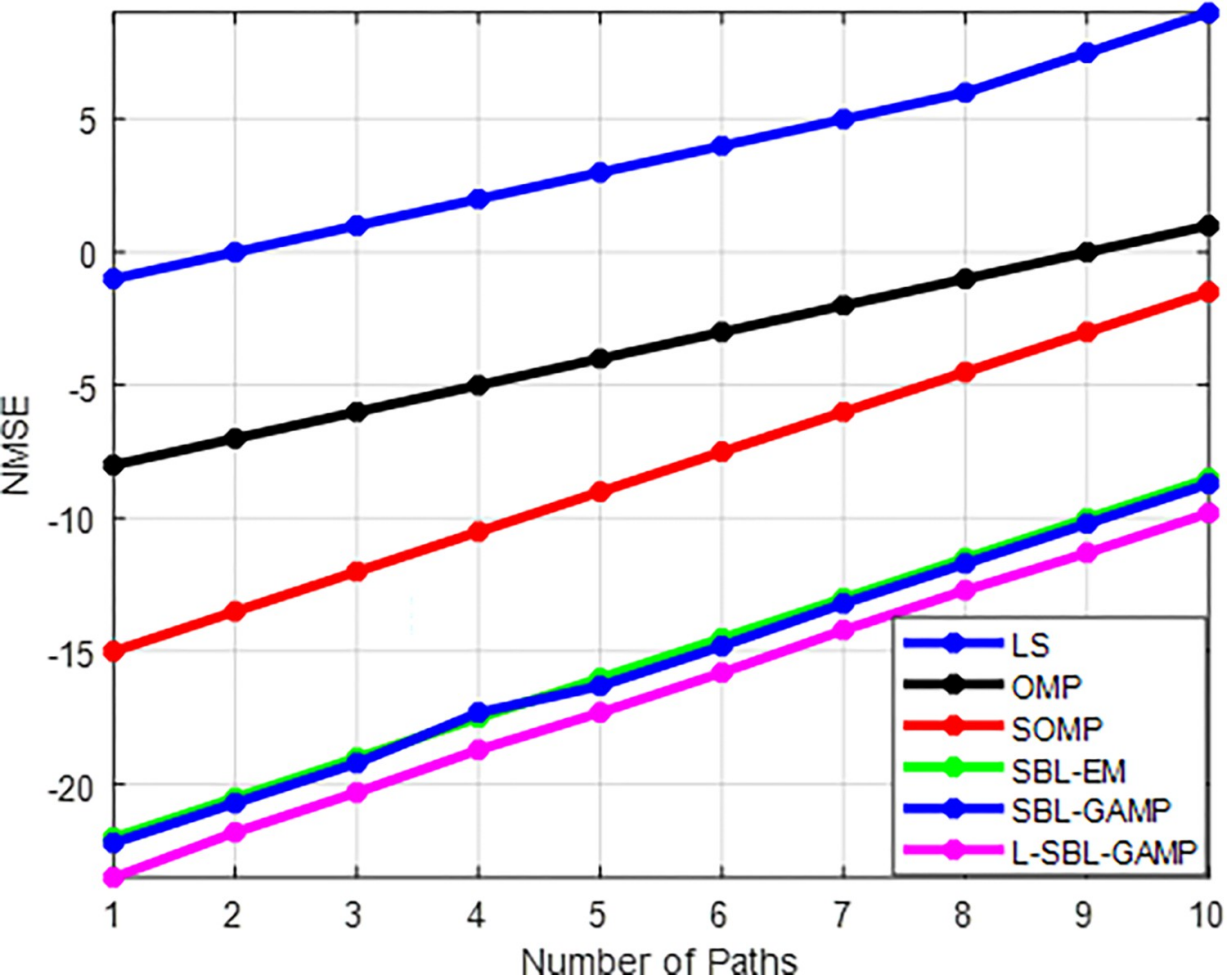
Number of path Vs NMSE.

Fig 5 shows the NMSE against the number of paths for the proposed algorithm. The path element L varies from 2 to 10. The large number of paths gives a high computational cost with high estimation error. This is due to the small neighboring angular distance. This leads to the uncertainty of adjacent points, which affects the estimation of hybrid precoder performance in mmWave systems. However, the proposed L-SBL-GAMP algorithm shows better performance compared to other existing algorithms.

**Fig 6:**
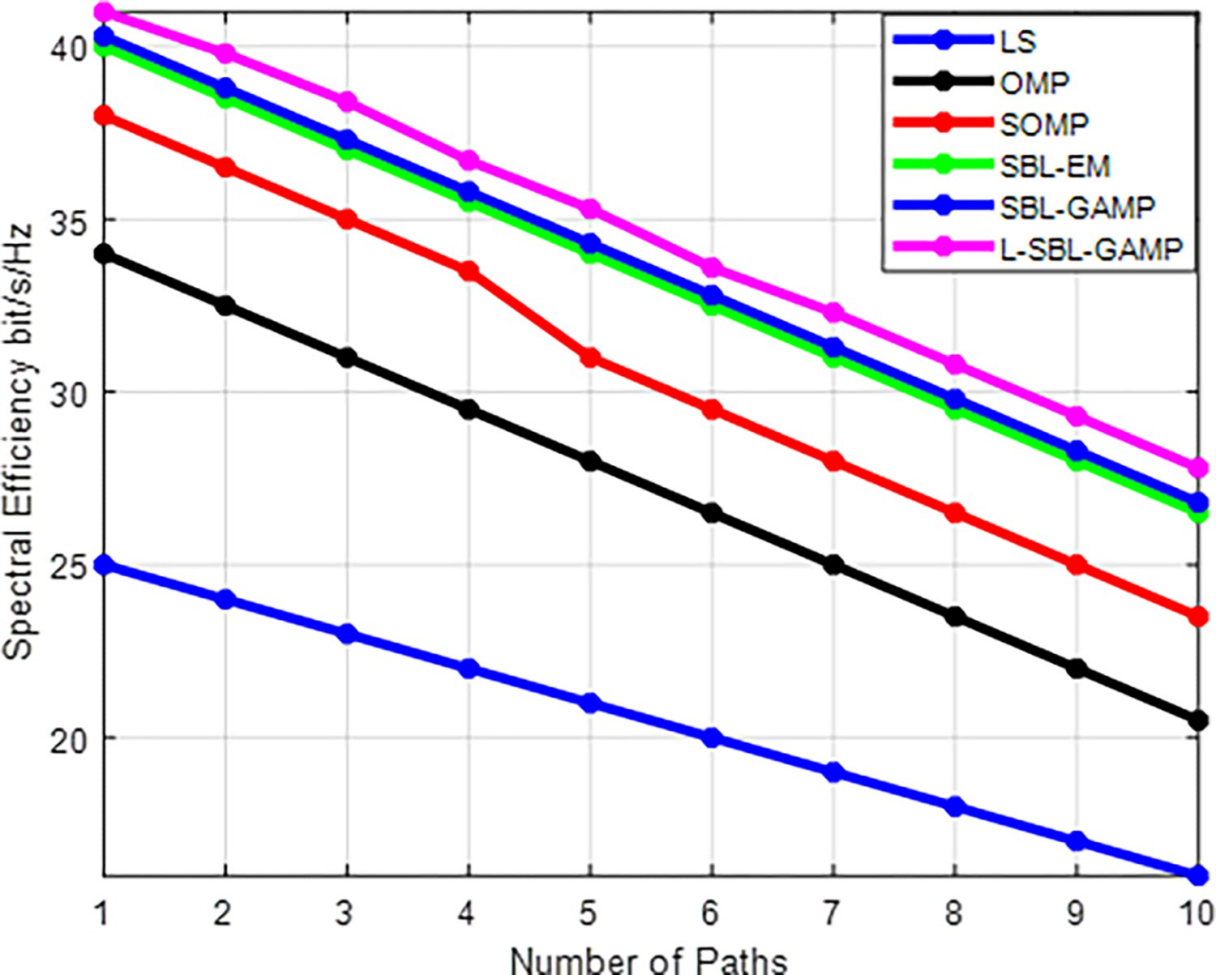
Number of path Vs spectral efficiency (bits/s/Hz).

The performance of the number of paths against the spectral efficiency with the proposed method is unveiled in Fig 6. The increased channel path decreases the spectral efficiency of the proposed algorithm. Moreover, increasing sparsity level leads to high errors in the precoder along with the high computational complexity in the mmWave channel.

**Fig 7:**
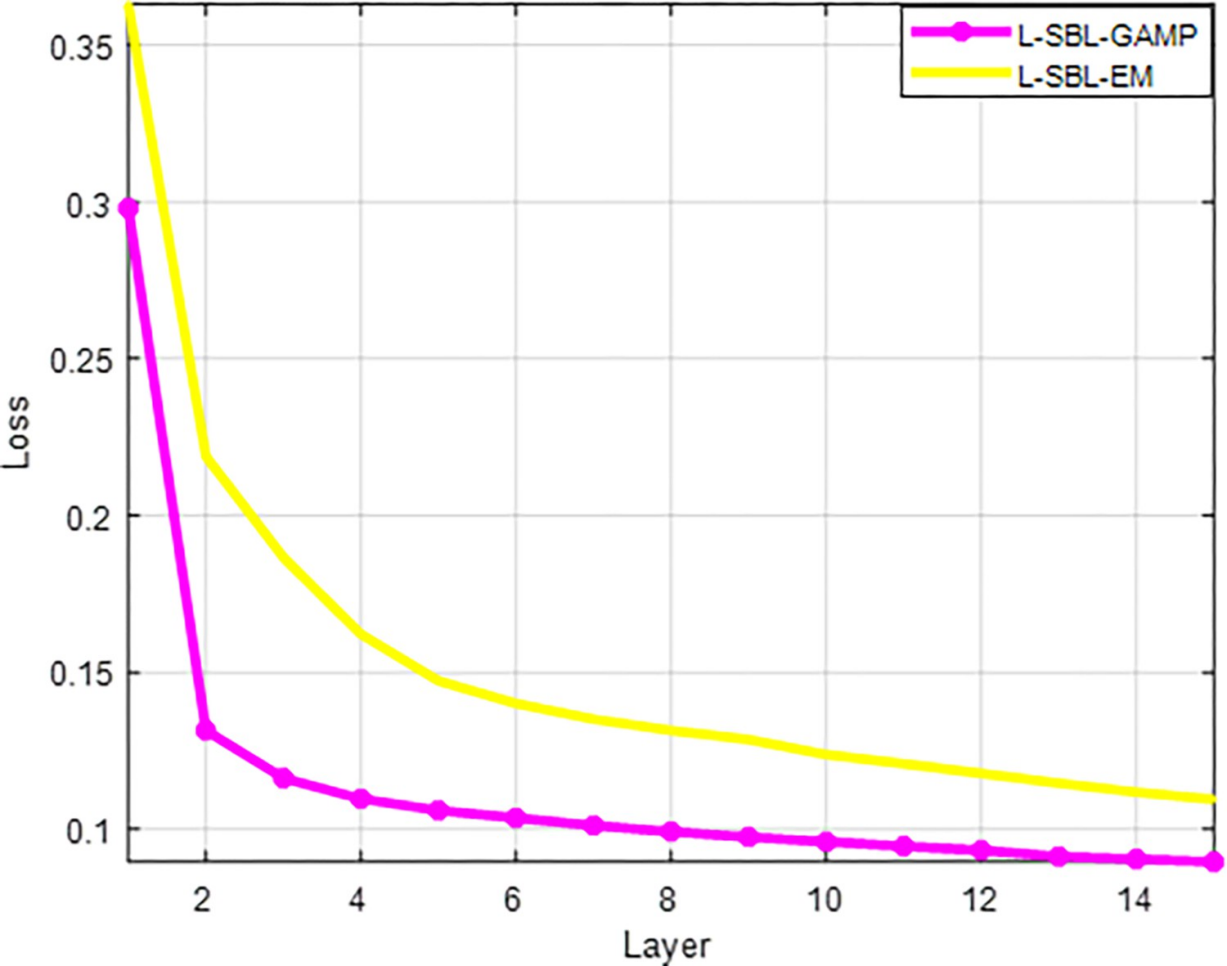
Layer Vs loss.

In Fig 7, the Layer vs loss function is discussed for L-SBL-EM and the proposed L-SBL-GAMP algorithm. The proposed L-SBL-GAMP gives a reduction in the loss function compared to the L-SBL-EM network.

**Fig 8:**
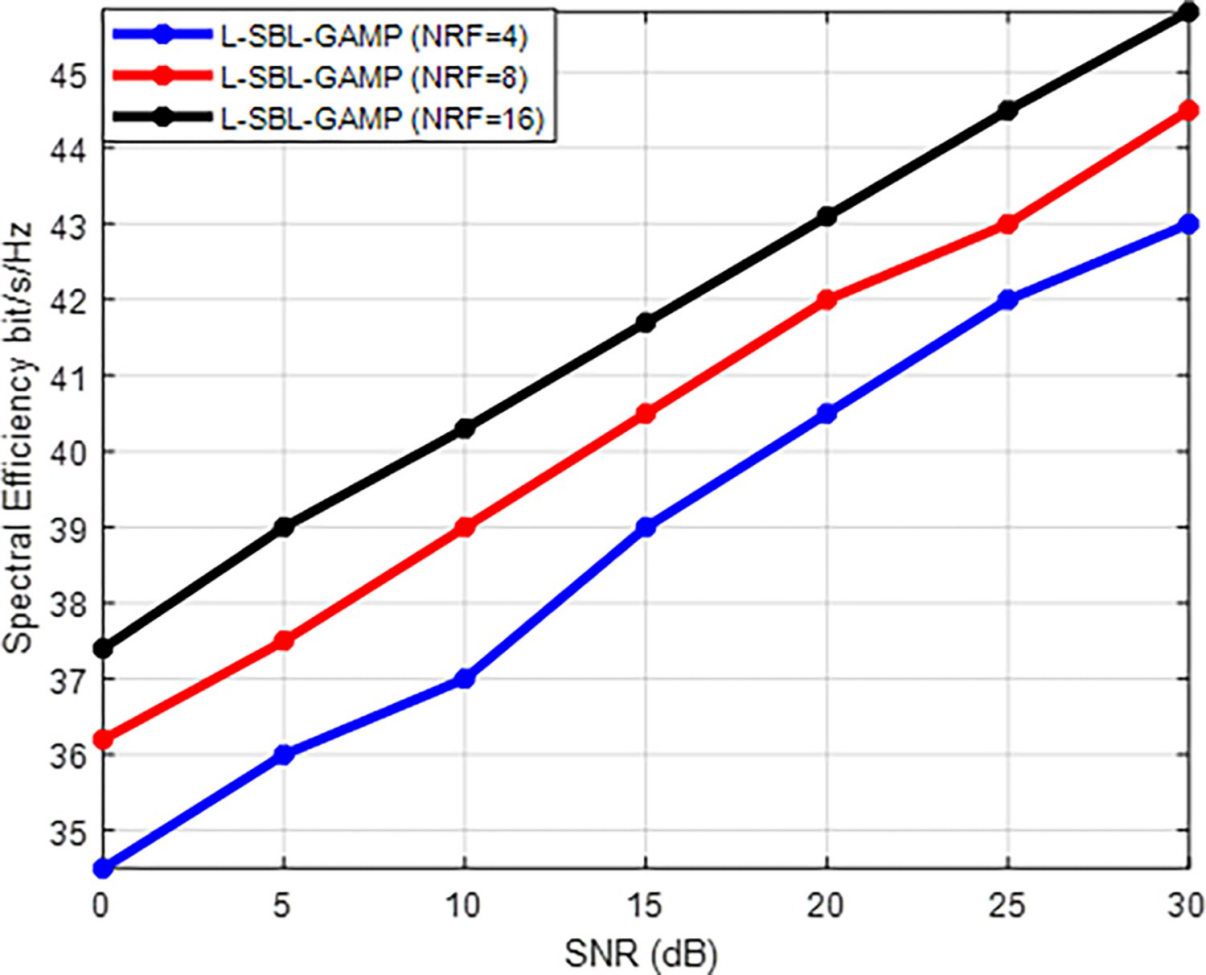
SNR Vs spectral efficiency (bits/s/Hz) for various RF chain.

**Fig 9:**
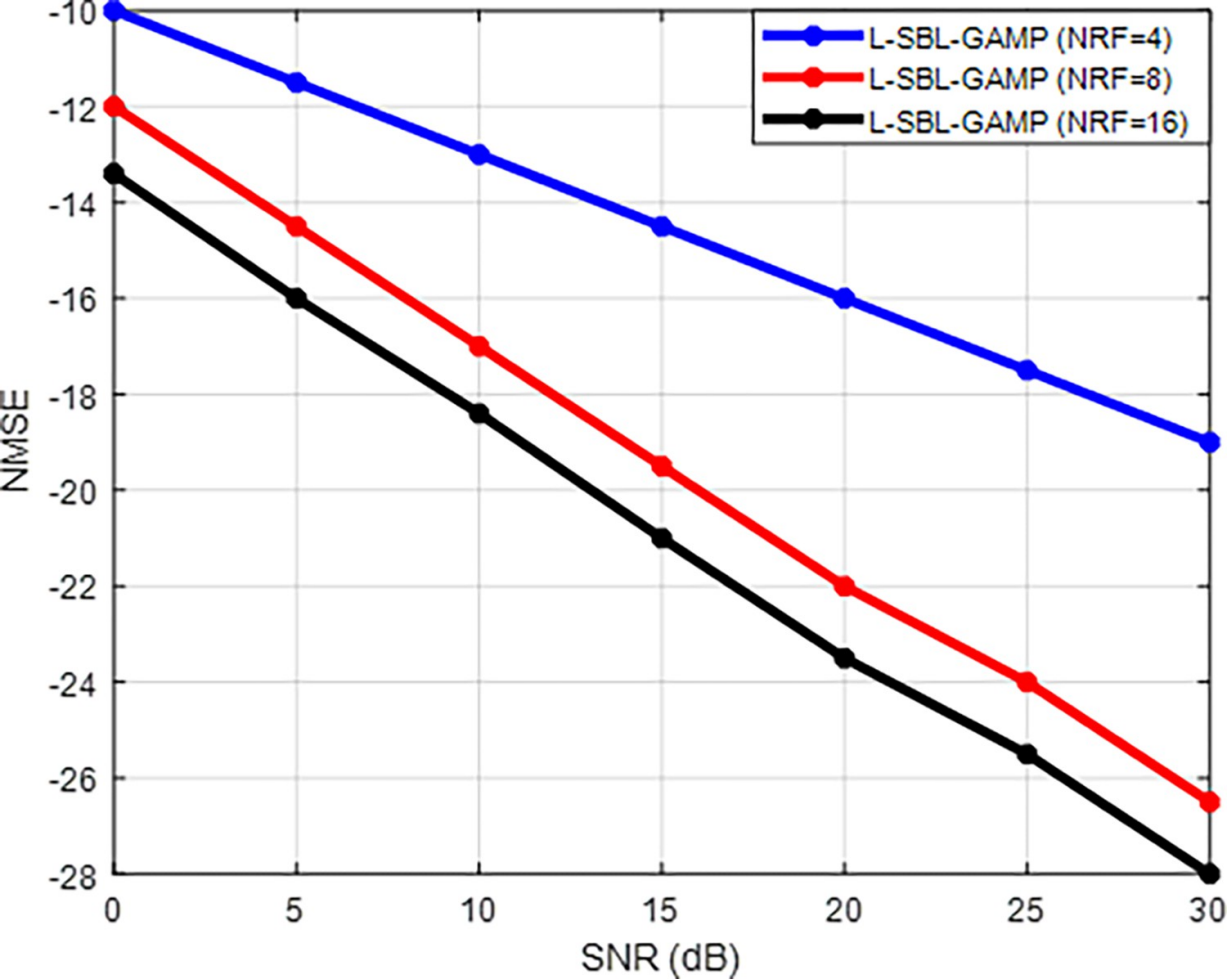
SNR Vs NMSE for various RF chain.

Figs 8 and 9 display the performance of the L-SBL-GAMP network concerning signal-to-noise ratio (SNR) versus spectral efficiency (SE) and SNR versus normalized mean squared error (NMSE) for varying numbers of RF chains. The results indicate that the proposed L-SBL-GAMP network achieves high spectral efficiency and superior accuracy as the number of RF chains increases. This improvement in spectral efficiency is due to the use of a larger set of random precoders, resulting in a decrease in estimation error through compressed sensing and an increase in the number of effective combining and precoding beam patterns. Consequently, it is preferable to use a larger number of RF chains to attain high spectral efficiency and reduce estimation error in mmWave systems. However, the use of a large number of RF chains increases the complexity of the mmWave architecture. Therefore, a trade-off between complexity and performance must be considered for real-time implementations.

**Fig 10:**
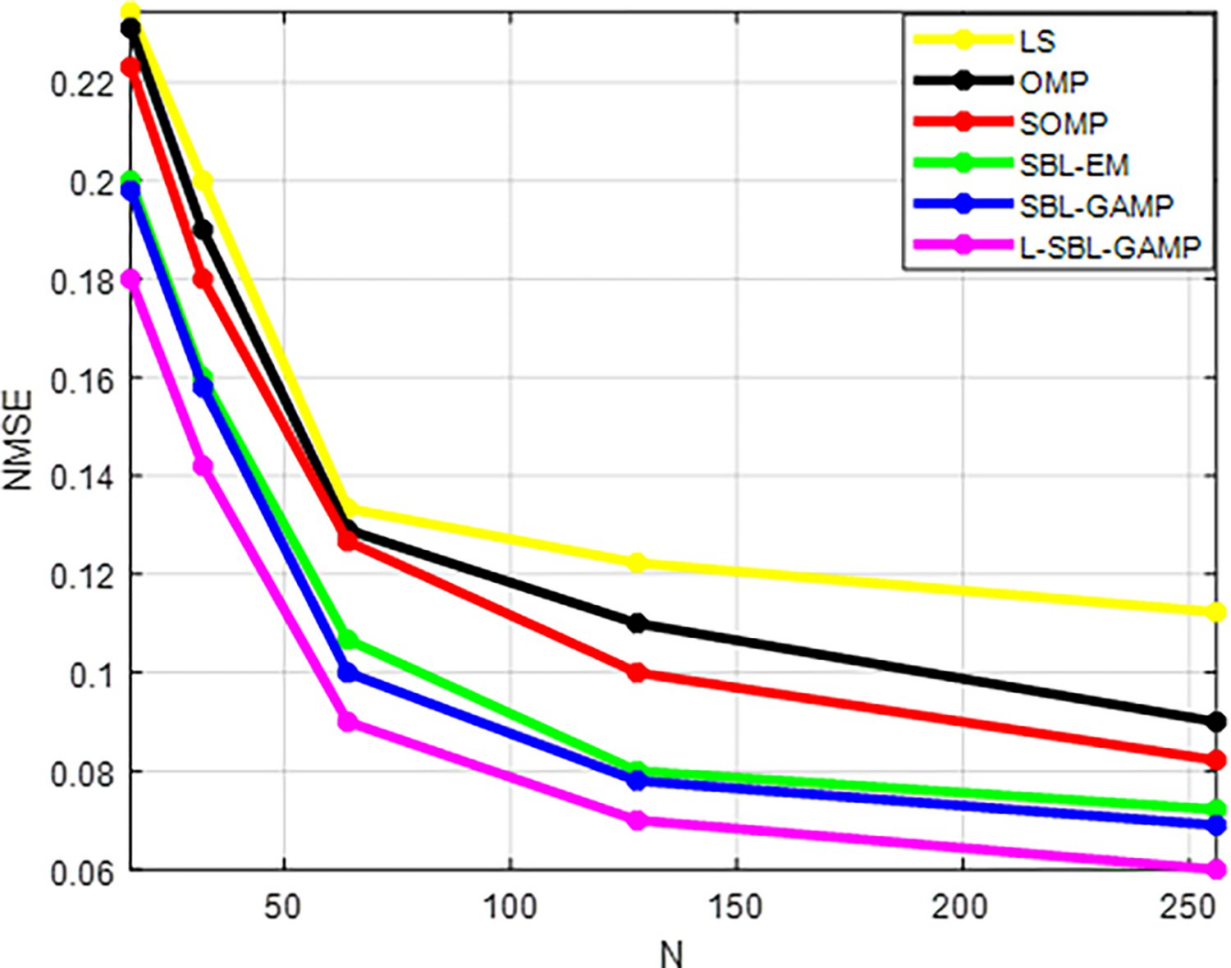
N Vs NMSE.

The curves depicting the normalized mean squared error (NMSE) versus the number of antennas (N) for the proposed and existing approaches are presented in Fig 10. As N increases, the NMSE value decreases for both the proposed and existing algorithms. However, the least squares (LS) algorithm shows almost constant performance for different N values. The proposed L-SBL-GAMP network demonstrates superior performance compared to other approaches. Although the proposed method achieves better results with an increased number of antennas, there is a corresponding increase in computational complexity.

**Fig 11:**
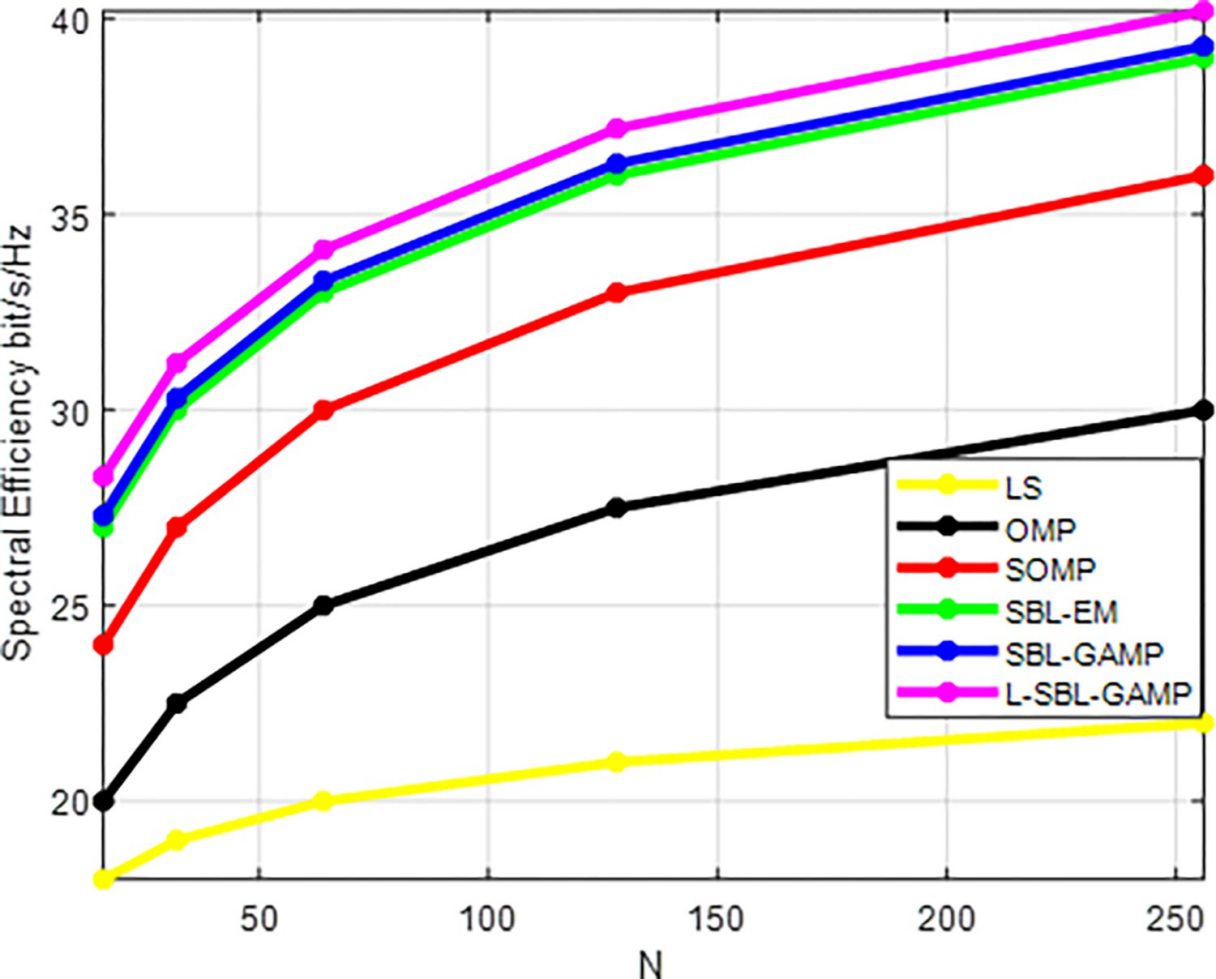
N Vs spectral efficiency (bits/s/Hz).

Fig 11 displays the number of antennas (N) versus the spectral efficiency for the proposed L-SBL-GAMP network and the existing method. the performance of the proposed L-SBL-GAMP network provides better spectral efficiency with an increase in the number of antennas for all hybrid systems.

## Conclusion

In this paper, a deep learning architecture named L-SBL-GAMP is proposed to design optimal hybrid precoders/combiner for the mmWave massive MIMO hybrid architecture. The proposed L-SBL-GAMP algorithm provides better spectral efficiency, accuracy, and low computational complexity compared to the SBL-EM method. In the proposed L-SBL-GAMP algorithm, we utilized all the T layers for the computation of hybrid precoders/combiners under the sparse nature of the mmWave channel. The experimental and the numerical results showed the effectivities of the proposed deep learning-based method, where it could be seen that the proposed deep learning-based algorithm worked better than the existing state-of-the-art methods, such as LS, OMP, SOMP, SBL-EM, and SBL-GAMP, and provided increased spectral efficiency and less Normalized Mean Square Errors (NMSEs).
